# Diffusion-Driven X-Ray Two-Dimensional Patterns Denoising

**DOI:** 10.3390/ma13122773

**Published:** 2020-06-18

**Authors:** Massimo Ladisa, Antonio Lamura

**Affiliations:** Istituto Applicazioni Calcolo—CNR, Via Amendola 122/D, 70126 Bari, Italy

**Keywords:** X-ray patterns, denoising, diffusion equation

## Abstract

The use of a mathematical model is proposed in order to denoise X-ray two-dimensional patterns. The method relies on a generalized diffusion equation whose diffusion constant depends on the image gradients. The numerical solution of the diffusion equation provides an efficient reduction of pattern noise as witnessed by the computed peak of signal-to-noise ratio. The use of experimental data with different inherent levels of noise allows us to show the success of the method even in the case, experimentally relevant, when patterns are blurred by Poissonian noise. The corresponding MatLab code for the numerical method is made available.

## 1. Introduction

The study of the properties of crystalline materials mainly relies on the use of X-ray diffraction (XRD) techniques, which give relevant information for several applications as, for example, in nanotechnology [1,2]. The processing of such data requires a chain of preliminary steps aiming at improving the quality of XRD data [3,4]. The removal of inherent noise is of fundamental importance since it helps in separating the effective crystallographic data from the background signal in order to estimate nano and biomaterial features. Several techniques were developed to reduce noise to acceptable levels [5,6,7,8,9,10,11,12,13,14,15,16]. Initial methods were based either on polynomial filtering of recorded spectra in spatial domain [5] or on the use of Fourier smoothing in frequency domain in order to remove the high-frequency components of the spectrum [17]. Other approaches were also proposed which make use either of the singular value decomposition [7,8] or of the Hankel-Lanczos singular value decomposition algorithms [10].

In the framework of image processing [18] a different approach was proposed by considering the diffusion process in order to filter images. The idea behind is that the diffusion equation applied to an image can smooth the noise. The main drawback is that the edge of images cannot be maintained since the Laplacian operator of the diffusion equation is linear. This aspect was conveniently overcome when anisotropic diffusion was proposed aiming to preserve the edge efficiently [19]. Indeed, the latter method is able to detect edges without smoothing and/or shifting them at the scale of interest. What the method makes is to iteratively filter the image by using a smoothing kernel with small support thus obtaining a sequence of diffused images with decreasing resolution. This is achieved by introducing an effective diffusion constant which depends inversely on the modulus of the image gradient. The net effect is to obtain a diffused image which converges to a final configuration without sharp edges. A further improvement was obtained by introducing a somewhat biased anisotropic diffusion [20] where the diffusion equation is supplemented by an additional source term which favors the convergence of the method to the solution of interest.

Several methods were later put forward. We cite, among others, non-local algorithms [21] and nonlinear scale-space approaches [22]. In the former case a procedure which relies on a non local averaging of all the pixels in the image, is introduced. The provided results show better performance in noise reduction with respect to local smoothing methods (like, for example, the ones in [19,20]) but the computational cost and complexity to be paid are relevant. This latter aspect is the main drawback of even more complicated formulations. Indeed, the results in [22] are very good but the method reveals to be complex in two spatial dimensions requiring the tuning of a total of four free parameters. The method which we propose to use for XRD data is very simple being local and with only one free parameter for two dimensional patterns, as later shown. The overall performance in terms of figure-of-merit indicators might be not the optimal one but this is a reasonable price to be paid when processing large set of images obtained in X-ray experiments. The rapidity and easiness in the processing chain are as well relevant to be taken into account. This does not preclude the possibility of further improvements [23].

The methods in [19,20] were devised to accomplish with the task on Gaussian-noise affected images, where mean and variance are not related each other; on the other hand, in several experimental setups the signal is recorded onto counting devices, such as photomultipliers, γ-counters, proportional chambers, imaging plates, charge-coupled-devices, (e.g., in XRD [24] or micrographs in cryo-electron microscopy experiments [25]), where a Poisson statistics is understood. It is known that a Poisson statistics resembles a Normal (Gaussian) one in the large counting limit (The probability density function for a Poisson distributed random variable reads:

$$f\left(n;\nu \right)\;=\;\frac{\nu^{n}e^{-\nu }}{n!}\;=\;e^{n\ln\nu -\nu -\ln n!}\;\approx \;\cdots \ln n!\;\overset{n\to +\infty }{\approx }\;n\ln n+\frac{1}{2}\ln n-n+1\cdots \;\overset{\frac{\nu -n}{n}\to 0}{\approx }\;\frac{\sqrt{2\pi }}{e}\;\frac{1}{\sqrt{2\pi }\sqrt{n}}\;e^{-\frac{{\left(\nu -n\right)}^{2}}{2{\left(\sqrt{n}\right)}^{2}}}\;,$$

i.e., a Gaussian probability density function ($\frac{\sqrt{2\pi }}{e}\approx 1$) with mean *n* and variance *n*, q.e.d.). In the present paper, we propose for the first time the use of the biased anisotropic diffusion equation [20] to XRD images showing the possibility of using it as a powerful method in the pre-processing of such images for Poisson noise reduction. We prove that the present method is simple and computationally efficient accomplishing its scope. A finite-difference method (FDM) is adopted to solve numerically the aforementioned equation. The paper is organized in the following way. In the next section we outline the mathematical model used and the numerical procedure to solve the diffusion equation. Then we present the results obtained when processing images with either Gaussian or Poissonian noise. Finally, we discuss the main findings of this work.

## 2. The Model

In this section, we present the model used to derive the anisotropic diffusion equation and the method to solve it.

Any image has an intensity level that can be represented as a function f:D→[0,1] where *D* is an open two-dimensional domain. Several methods were proposed that use the minimization of a proper energy functional to obtain a reconstructed image *u* starting from the degraded image *f*. Here we will consider the functional proposed in Ref. [20] which proved to be efficient in image filtering [26]. It reads as

(1) $$E_{f}\left(u,w\right)={\;\int \int \;}_{D}\;\alpha \left[\beta {\left(u-f\right)}^{2}+w{\left|\nabla u\right|}^{2}+\lambda^{2}\left(w-\ln w\right)\right]d^{2}x$$

where dimensional parameters α,β, and λ are non-negative and w:D→[0,1] is a continuity control function such that for each reconstructed image *u*, the cost $E_{f}$ has its minimum for one optimal control function $\bar{w}$. The resulting partial differential equation is obtained from the minimization of Equation (1) by using the maximum principle [27] and reads as [20]

(2) $$\partial_{t}u=\nabla \cdot \left(\alpha w\nabla u\right)+\alpha \beta \left(f-u\right)$$

where the function *w* is chosen to be $w=1/(1+|\nabla u{|}^{2}/\lambda^{2})$ [20]. Equation (2) is a diffusion equation with diffusion constant αw supplemented by a source term αβ(f−u). It is useful to cast it in dimensionless form

(3) $$\partial_{\tau }u=\nabla_{s}\cdot \left(w\nabla_{s}u\right)+\left(f-u\right)$$

where the new variables τ=αβt and $s=\sqrt{\beta }x$ are dimensionless being $\left[\alpha \right]=\left[L^{2}/T\right]$ and $\left[\beta \right]=\left[1/L^{2}\right]$.

The diffusion Equation (3) is solved by using a finite-difference scheme [28]. The function *u* is defined on the nodes of a square lattice with spacing $\Delta s=\sqrt{\beta }\Delta x$. The variable τ is discretized in steps Δτ=αβΔt with values $\tau_{n}=n\Delta \tau$, n=1,2,3,.... Any discretized function at time $\tau_{n}$ on a node ($x_{i},y_{j}$) (i=1,...,L;j=1,...,L) of the lattice of size L×L is denoted by $h\left(x_{i},y_{j},\tau_{n}\right)=h_{ij}^{n}$. The diffusive term is implemented by using an explicit Euler algorithm as

(4) $$u^{n+1}=u^{n}+\Delta \tau \left[\nabla_{s}\cdot \left(w^{n}\nabla_{s}u^{n}\right)+\left(f^{n}-u^{n}\right)\right]$$

where the derivatives $\nabla_{s}$ are computed by using a second-order centered scheme

(5) $$\nabla_{s}h_{ij}^{n}=\left(\frac{h_{i+1,j}^{n}-h_{i-1,j}^{n}}{2\Delta s},\frac{h_{i,j+1}^{n}-h_{i,j-1}^{n}}{2\Delta s}\right)$$

The degraded image *f* is used as initial condition. We set Δx=Δt=1 and the value of λ in the function *w* was fixed to be 0.9∑|∇u|/C where C=L×L is the resolution of the image [29]. The parameter α was varied to guarantee the numerical stability of the numerical method while β was used to optimize the procedure for getting the best improved image in the sense that will be later discussed.

## 3. Numerical Results

In this section, the results in the processing of different kinds of images will be presented.

The only parameters that are left in the numerical solution of Equation (3) are α and β. We found that α=0.1 is the highest value that can be used to guarantee stability without slowing down the convergence of the method to the optimal solution. Indeed, one has to balance between the need for using the highest possible value of Δτ to reduce the number of iterations to achieve the final reconstructed image and the requirement of stability which fixes an upper bound for Δτ. The efficiency in image filtering is evaluated by measuring the peak signal noise ratio (PSNR) defined as

(6) $$\mathrm{PSNR}\overset{def.}{=}-20\log_{10}\left(\frac{\sqrt{\langle |u-f_{0}{|}^{2}\rangle }}{\max(f_{0})}\right)\;,$$

where $f_{0}$ is the original image without noise and 〈...〉 denotes a sum over all the pixels of the images. This quantity is measured in decibels and higher values correspond to a better denoising. The parameter β was varied in our method to obtain the highest possible values of PSNR.

**Figure 1:**
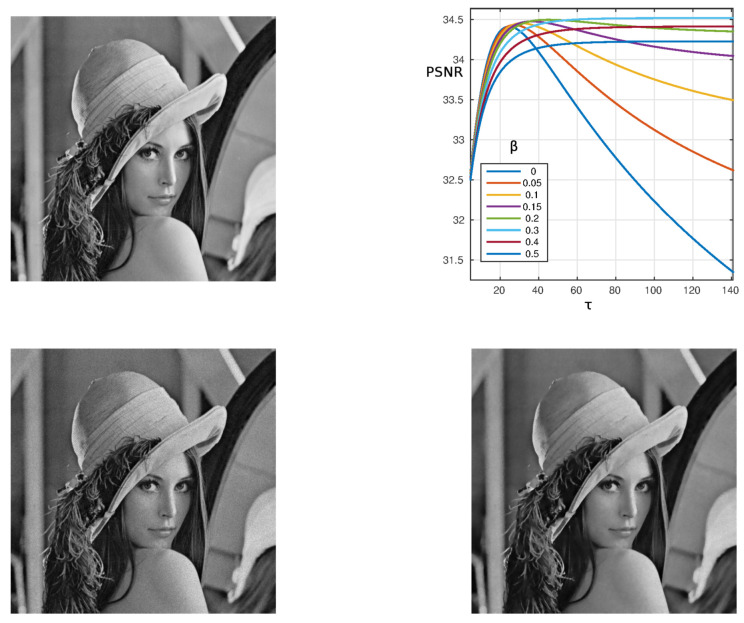
Algorithm performance for the *Lenna* image with resolution 512×512. **Top left**: Noiseless image $f_{0}$; **Top right**: PSNR as a function of τ for α=0.1 and β=0−0.5. **Bottom left**: Input noisy image *f* with 5% Gaussian noise; **Bottom right**: Best output image for β=0.3.

We first tested the method on the classical *Lenna* image where Gaussian noise with zero average and variance 0.05 is added on the noiseless image $f_{0}$ with resolution 512×512. The results are shown in Figure 1. It can be seen that for β≤0.2, the PSNR decreases at high values of τ after reaching a maximum. For values β>0.2 keeps constant after an initial increase. The final value decreases with the increase of β. The best performance, in terms of the PSNR, is obtained for β=0.3 and the corresponding final reconstructed image is reported in the bottom right panel of Figure 1.

**Figure 2:**
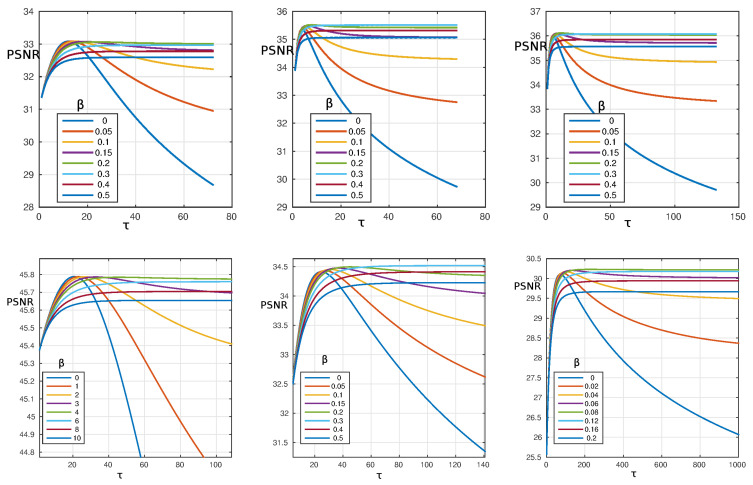
**Top**: PSNR as a function of τ for different resolutions of the *Lenna* image. **Left**: α=0.1, β=0−0.5 and resolution 171×171; **Middle**: α=1, β=0−0.5 and resolution 768×768; **Right**: α=1, β=0−0.5 and resolution 1024×1024. **Bottom**: PSNR as a function of τ for different noise level of the *Lenna* image (resolution 512×512). **Left**: α=0.01, β=0−10 and noise 0.01; **Middle**: α=0.1, β=0−0.5 and noise 0.05; **Right**: α=0.1, β=0−0.2 and noise 0.1.

In previous studies [26], where Equation (2) was solved by using a different mathematical method, the role of the image resolution on the PSNR was not investigated. To fill this gap we repeated the aforementioned procedure of Figure 1 by changing the resolution of the processed image. The qualitative behavior of the PSNR is similar for the different resolutions a shown in Figure 2. However, some comments are in order. For the lower resolution (171×171) the best output is obtained for β=0.2 while better resolutions (768×768 and 1024×1024) have the optimal performance for β=0.3 which is the same values found in Figure 1. This indicates that when increasing the resolution of the image, the best value for β does not change (To keep the correct scale with respect to the original image (*L* = 512) the β value was preliminarily rescaled, i.e., β→β×512/L). On the contrary, the final value of the PSNR increases when improving the resolution.

**Figure 3:**
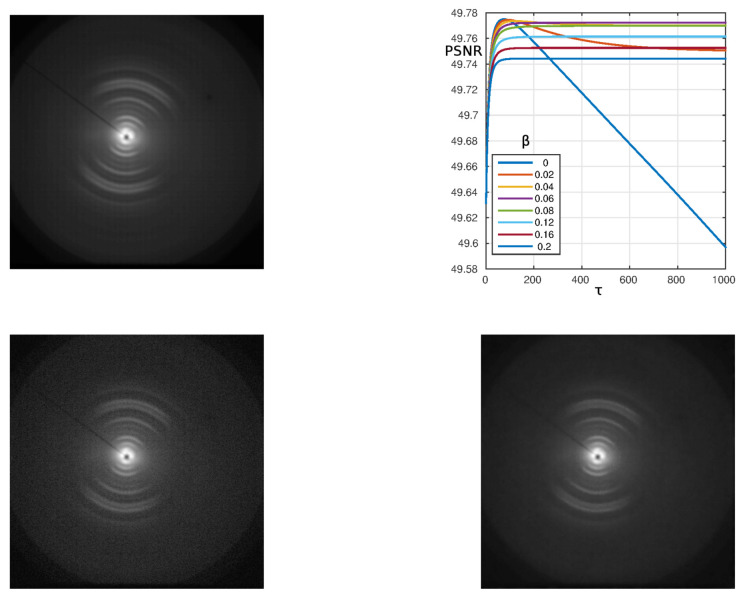
Algorithm performance for the XRD image with resolution 512×512. **Top left**: Noiseless image $f_{0}$ (rescaled to the input image); **Top right**: PSNR as a function of τ for α=0.1 and β=0−0.2; **Bottom left**: Input image *f* with Poissonian noise; **Bottom right**: Best output image with β=0.06.

Beside the Gaussian-noise affected images, the experimental setups often collect data on two-dimensional arrays, where a Poisson counting statistics accordingly needs a noise reduction. In order to investigate the present approach on such noisy data, we applied the model to denoise XRD patterns collected on the collagen molecules in tendon-derived collagens, where under several biochemical conditions, a super-organization of tissue into triple helices (with a preferred orientation displayed by the π-symmetric partial arcs of Figure 3 replacing the fully 2π-symmetric circles) and a high crystalline domain can trigger mechanical stiffness (for details on sample preparation and experimental setup the interested reader is addressed to [30]). In Figure 3 the XRD patterns are shown. The noisy image (bottom left panel) was integrated on a collection time of 2400 s, resulting in a maximum counts below 1000, while the noiseless one (top left panel) required 327,350 s, with maxima reaching some 60,000. The input image was processed for several values of the parameter β. The results for the PSNR are illustrated in the top right frame of Figure 3. The PSNR has trends similar to the case of Figure 1 with Gaussian noise. It can be seen that for β≤0.04, the PSNR decreases in the limit of high τ after reaching a maximum. When β>0.04, the PSNR stays constant after an initial increase. The final value decreases when increasing β. The output image with the best PSNR, obtained with β=0.06, is reported in the bottom right panel.

The optimal values of β in maximizing PSNR depend on the type of noise. They are reported in Table 1 where we also compare the efficiency in noise reduction for the images with Gaussian (different levels) and Poissonian noises considered in the present work. The final values of PSNR strongly depend on the type of noise being higher in the case of XRD patterns.

**Figure 4:**
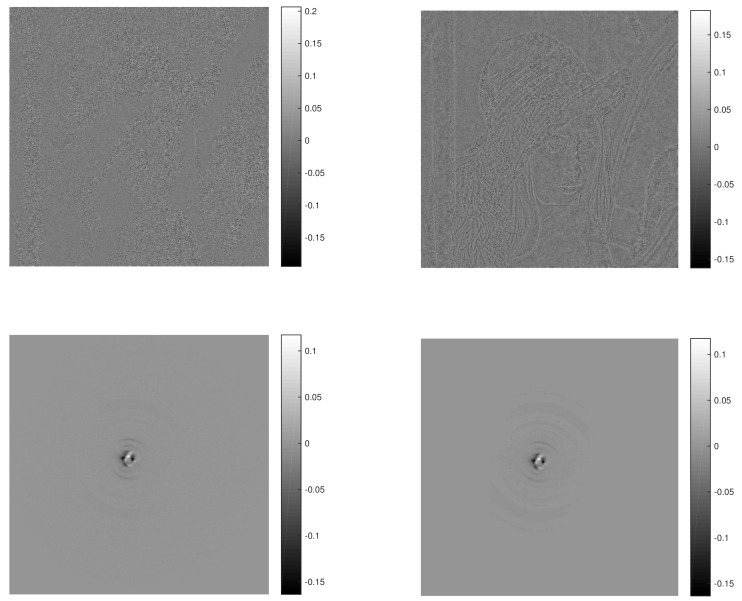
Difference-images with respect to the noiseless 2D pattern for the initial (noisy, on the **left**) and final (denoised, on the **right**) images with resolution 512×512. The colorbar shows the difference ratios with respect to the noiseless pattern. **Top**: Lenna (0.05 Gaussian noise level); **Bottom**: XRD pattern (Poissonian noise).

To stress the algorithmic efficiency, we added the values of another figure of merit, namely the structure similarity (SSIM), as defined for instance in [31] and implemented in MatLab: PSNR/SSIM values increase for all cases analysed in this paper; they are in agreement with the visual inspection of the difference-images before/after applying the algorithm, as reported in Figure 4.

## 4. Discussion and Conclusions

We proposed the use of a denoising procedure for X-ray diffraction patterns. The method, which is based on the numerical solution of a generalized diffusion equation, was never been tested and adopted for XRD images which are of relevant experimental interest. Such images are characterized by the presence of Poissonian noise, typical of patterns obtained by counting detectors. The processing of such images is completely different from those affected by Gaussian noise due to the very different inherent nature of the noises, as previously commented. For this reason the elaboration of the former patterns required in the years devoted approaches. The main limitations in the available studies are related either to the computational cost [11] or complexity [14]. Very recent algorithms were proposed to tackle this problem for high-energy X-ray patterns [16] or for X-ray medical images [15].

The proposed method is characterized by a significant simplicity since the Equation (3) can be easily implemented by, for example, a MatLab code. Despite of this, it can attain significant performance even in this case by reducing the noise level as witnessed by the measured values of the peak signal-to-noise ratio and of the structure similarity. In particular, we showed that an improvement in the values of the PSNR and of the SSIM can be obtained when considering also the source term (β≠0) in Equation (2). The parameter β is the only free parameter that has to be tuned to obtain the better performance of the method. The advantage in using an effective diffusion constant, which depends inversely on the modulus of the image gradient, is to obtain a final image without sharp edges while the source term facilitates the convergence of the algorithm.

The present results open up the possibility of using the method in the pre-processing chain of X-ray diffraction images or cryo-electron microscopy micrographs. Moreover, the MatLab code developed to implement the method is computationally efficient to allow the processing of a high numbers of images in reasonable amount of time. As a matter of comparison we report that the processing-time for a typical XRD pattern is of a few seconds on a laptop. The availability of several denoising methods asks for an intensive and accurate comparison among them. We devote this study to a future publication.

## Figures and Tables

**Table 1:** Improvement of the PSNR and SSIM values with the use of the denoising procedure in the cases of images (C=512×512) with Gaussian (*Lenna*) and Poissonian (XRD) noise with the corresponding values of β.

Image	β	Noise	PSNR (Initial)	PSNR (Final)	SSIM (Initial)	SSIM (Final)
Lenna	4	0.01	45.31	45.77	0.93	0.94
Lenna	0.3	0.05	31.33	34.52	0.59	0.64
Lenna	0.08	0.10	25.31	30.22	0.41	0.46
XRD	0.06	−	49.61	49.77	0.10	0.33
